# Implementation of mHealth applications in community-based health care: Insights from Ward-Based Outreach Teams in South Africa

**DOI:** 10.1371/journal.pone.0262842

**Published:** 2022-01-25

**Authors:** Ronewa Suzan Tshikomana, Mokholelana Margaret Ramukumba

**Affiliations:** 1 Department of Social Development, Makhado, Limpopo Province, South Africa; 2 Department of Health Studies, University of South Africa, Pretoria, Gauteng Province, South Africa; South African Medical Research Council, SOUTH AFRICA

## Abstract

**Background:**

Ward-Based Outreach Teams’ (WBOTs) use of mobile technologies can promote better quality and improved health services for populations in low- and middle-income countries. However, the implementation of such systems is fraught with threats to sustainability.

**Purpose:**

The purpose of this study was to gain a deeper understanding of users’ views and experiences of the implementation of mHealth in a selected sub-district in North West Province, South Africa.

**Methods:**

The study was qualitative, exploratory and descriptive. Data were collected from community health workers (CHWs) (n = 24) and outreach team leaders (OTLs) (n = 4) who used mobile devices loaded with the Mobenzi application through semi-structured focus group interviews, using an interview guide. Creswell’s stages of thematic analysis were used for data analysis, and codes, categories and themes were validated with the participants.

**Results:**

Three themes emerged from the findings, namely the transition from a paper-based system to an electronic system; the use of the application; and reverting to paper-based documentation. The findings revealed that WBOTs considered the mHealth application as useful, easy to use, and supportive to their workflow. They experienced some technical challenges and lamented the inaccessibility of technicians. The mobile initiative’s termination caused distress among the participants, especially CHWs who had to revert to paper-based documentation to capture community health data. OTLs were concerned about the quality of health data being captured in the absence of the application.

**Conclusions:**

The study concluded that community-based health care programmes that use WBOTs and mHealth technologies are essential in strengthening the health care system. WBOTs are facilitators for improving access to health care. Policy-makers and district managers will gain useful insights if they engage application users in discussions regarding future mobile health interventions for household and community-based care.

## Introduction

South Africa has established and re-engineered a new primary health care (PHC) model that integrates care across different levels of the health care system, including community-based care, where health care is taken to homes and communities [1, 2]. This involved shifting tasks and care responsibilities from physicians and nurses to trained community health workers (CHWs) who assist with specific health care services [3]. Health practitioners are currently reaffirming the importance of recruiting and retaining CHWs in order to achieve key public health goals [4]. CHWs have become a prominent component of Ward-Based Outreach Teams (WBOTs), now called Ward-Based Primary Health Care Outreach Teams (WBPHCOTs).

To strengthen the teams, the Department of Health developed mHealth information technologies to support their roles and responsibilities [5]. The mHealth applications designed for WBOTs were utilised to conduct household profiling, enrol clients, screen diseases, refer clients and manage monthly reports [6]. In selected sub-districts in this province, one core activity for CHWs was to collect primary health data from clients in communities using the mHealth application–called Mobenzi–for team leaders’ consolidation on the District Health Information System (DHIS). The system’s implementation represented a significant paradigm shift in WBOTs’ tasks and was conceptualised as empowering existing CHWs with smartphone-based tools [7]. The North West Province was among the few provinces within which mHealth programmes were piloted [7]; other provinces in the country used different applications for the WBOTs.

Odendaal and Lewin posit that mobile phone interventions have proven beneficial in terms of rapid data collection, storage, and general health information management [8]. However, collected data must be complete, accurate and comprehensive in order to positively contribute to data-driven decision-making in health care. Also, users need to see potential in mobile technology. In South Africa, mHealth is perceived as having significant potential to benefit health service delivery processes, especially in resource-constrained areas [9]. The South African mHealth strategy of 2015 acknowledges the need to align mHealth with organisational objectives and end-user needs, paying attention to issues of usability and security of information [1]. Therefore, end users’ experiences of the mHealth intervention are some of the areas identified for further research to enable policy-makers to collect evidence on the need to roll out future mobile health technology in other districts within the province and country.

Despite recording some significant improvements since independence in 1994, the South African health care system still faces a number of health challenges [10]. The challenges affecting the implementation of mHealth in the country include a lack of alignment and integration of the interventions into health plans; the absence of government leadership and coordination; poor documentation and learning from best practices; a lack of interoperability; and the absence of a single framework within which to evaluate the role of mHealth and eHealth tools to strengthen the health system [11]. Moreover, mHealth devices are constrained by small screen sizes and available interaction mechanisms [12].

Many of the mHealth barriers also go beyond the mobile technology itself; they are related to broader health system challenges in the health personnel’s practices, the integration of new technology with existing information systems, sustainable funding, and appropriate leadership to steer these shifts [5]. Despite growing demands, Modi and Mohanty argued that mHealth solutions come with their own set of challenges, including data confidentiality, security and safeguarding personal data. These are evident concerns in the health care industry, particularly since compliance with the Health Insurance Portability and Accountability Act is essential [13]. Moreover, most of the mHealth interventions were initiated with the help of donor funding, which may pose a challenge for sustainability in the long term [14].

The purpose of this study was to gain a deeper understanding of users’ views and experiences of the implementation of mHealth in a selected sub-district in North West Province, South Africa. This paper reports on part of the larger study evaluating mHealth in four provinces.

## Methods

### Setting

The Dr Kenneth Kaunda District (DKK) in the North West Province was chosen as the research setting. In 2011, the Department of Health designated it as the National Health Insurance (NHI) pilot district for the North West Province; mHealth projects were implemented in NHI pilot sites. The district is the smallest in the province, with only three local municipalities/sub-districts, and of these, only two municipalities/sub-districts implemented the mHealth application (Mobenzi). The Maquassi Hills sub-district health services–the research site–consist of one district hospital, two community health centres and six PHC facilities. The sub-district has a significant rural population, which may have an impact on patients accessing health care [15]. Thirteen WBOTs were responsible for this population, and interviews were conducted at two pre-arranged venues near the clinics to which the CHWs were linked. CHWs did not have designated areas attached to the health facility for their exclusive use, each facility arranged a meeting place for them, which could be a couple of blocks away.

### Population and sampling

The population consisted of the WBOTs, namely the CHWs and outreach team leaders (OTLs) who used mHealth to perform their daily tasks. The CHWs (n = 24) and OTLs (n = 4) were all professional nurses. The eligibility criteria were WBOTs who were registered professional nurses, trained on the Mobenzi application, and who had used Mobenzi for six months or longer. The study used purposeful typical case sampling to obtain typical cases of mHealth users. Specific criteria were also set for recruitment, and only individuals who were contracted by the Department of Health were included in the research sample. Individuals operating under the Department of Social Development were excluded, as they received different training and the mHealth application was mainly the responsibility of the health ministry.

After the province’s Department of Policy, Planning, Research, Monitoring and Evaluation provided permission to the team to conduct the study, MMR contacted the WBOT manager to introduce the main study. MMR sent the information leaflet, consent forms, as well as the preliminary interview guide to the WBOT manager through email. The manager provided a list of all 51 WBOTs in the sub-district and wards in which they operated. MMR then visited the sub-district to provide more clarity to the WBOT manager of the research purpose, the process of the study, and selection criteria. WBOTs linked to the two community health centres and six PHC facilities were invited to participate in the study, and 40 team members met the eligibility criteria.

### Data collection

Semi-structured focus group interviews were conducted with 28 available WBOTs over two groups. The WBOTs who met the inclusion criteria and who were willing to participate were given consent forms that explained the circumstances of participating in the study. MMR, the principal investigator, explained the nature of the research, procedures, potential risks and benefits to them, reasons for the study, and how the collected information would be used, were explained. All participants agreed, signed the consent forms, and completed their biographical details on the form provided. Ground-rules relating to the use of mobile phones and group dynamics were discussed. RST, the lead interviewer, disclosed that she was pursuing graduate studies, and at the time of data collection, she was working in a PHC facility with community-based groups under the Department of Social Development in a different province. She was also familiar with and participating in the main mHealth project. MMR asked follow up questions.

Focus groups were particularly suited for this study because they are inexpensive, flexible, stimulating, cumulative, elaborative, and capable of producing rich data [16]. Babbie confirmed that focus group interviews are advantageous because they create meaning, allow the participants to engage in discussions, interact on a topic, articulate and share their ideas freely [17]. There was sufficient time for the interviewer to follow up on leads, probe, and ask questions for clarification when necessary during the focus group interviews [18]. An interview guide was developed to explore different areas in mHealth implementation, which allowed views to be compared between participants [16]. The grand tour questions were as follows: *What are your views regarding the implementation of Mobenzi in this area*? *How did you experience the mHealth application*? *How did the mHealth initiative end*? *What are the implications*? (S1 Table). Participants also had an opportunity to ask questions, and each interview lasted approximately 1 hour 30 minutes. An audio recorder was used to capture all data, which was later transcribed. The data were stored in adherence to local rules and policies regarding confidentiality and data security. No field notes were captured.

The interviews were conducted in English. However, the CHWs were allowed to speak in the language of their preference, and other participants and OTLs assisted with translation. RST planned key questions in advance, and kept them open to allow for more discussion, to verify interpretations by asking supplementary questions, and allow additions and corrections to avoid leading questions [16]. Interviews continued until no new information emerged. Data collection only commenced after ethical clearance was obtained from the university (HSHDC/759/2017), and permission to conduct the study was granted by the province. This study was part of a larger study, *Evaluation of mobile technologies utilized for rural healthcare workers in South Africa and Sweden* (SAMRC/FORTE-RFA-01-2016-Category 3).

### Trustworthiness

Trustworthiness refers to adherence to certain principles in the conduct of qualitative research [19]. To ensure trustworthiness in this study, Lincoln and Guba’s model (1985) was used to describe how credibility, dependability, confirmability and transferability were ensured. There was prolonged engagement with the participants, they could communicate in the language with which they felt comfortable, and dependability was ensured by double-checking the findings and verifying information with participants. Original transcripts and audio recordings were kept to ensure the information provided by participants was their own. Data were collected until data saturation occurred, and thick descriptions were provided to ensure transferability.

### Data analysis

Qualitative analysis begins at the onset of a study and continues throughout the research process [20]. This is a process that requires the exploration, organisation, interpretation and integration of research material in such a way that the researcher retrieves, rethinks, compares subsets, identifies patterns and relationships, while organising data in a more meaningful way [21]. Thematic analysis was carried out manually, and the codes and categories that emerged were organised into themes. The analysis was inductive and iterative. Thematic analysis was used as a way to understand and interpret the data that were generated [21]. To ensure the quality of the collected data, focus group discussions were recorded, transcribed and edited where necessary by RST. After reading all the interview transcripts, RST marked sections of data and assigned them codes or labels. These were categorised, summarised and grouped into themes that represented the views of the WBOTs. A table of themes, sub-themes and categories was created and discussed with MMR; this involved immersion into the data to verify the findings. Consistency was checked by an independent analyst affiliated with the project. The themes that emerged were cross-checked to derive meanings, and were aligned with the research questions and objectives [21]. The coding tree is attached as supplementary information. (S1 File. Coding Tree)

## Results

### Biographical information

All 4 WBOTs in the sub-district were invited to participate in the study. A total of 28 WBOTs, comprising 24 CHWs and four OTLs, participated in two focus group discussions. They represented four of the eight health facilities. Twelve participants were male, and 16 were female. The average age of participants was 37, and their average experience using the system was two years. Participants’ statements are included in the discussion to support the findings. The four OTLs’ statements are referred to as TL1-TL4, and quotes from the 24 CHWs are referred to as P1-P24. The WBOTs manager was present in both group discussions, but was a non-participant.

Three themes with related sub-themes and categories emerged from the data: the transition from a paper-based system to an electronic system (Mobenzi); the use of the application; and reverting to paper-based documentation. Table 1 gives an overview of the themes.

**Table 1:** Themes.

Themes	Sub-themes	Categories
Transition from a paper-based system to an electronic system	Evolution of the mHealth application Capacity building	• Need to improve data capture
		• Training and technical support
		• Improving flow of communication
Using the application	Experiences of using Mobenzi	• Effective monitoring and supervision
		• Decision support system
		• Challenges experienced
Reverting to paper-based documentation	Initial reaction and acceptance of the inevitable Data management difficulties	• Response to termination of mHealth
		• Impact of paper-based documentation

#### Theme 1: Transition from a paper-based system to an electronic system

*Need to improve data capture*. WBOTs who have been part of the health system since PHC’s re-engineering explained that they initially used paper-based recording methods, then transitioned to an electronic documentation system. They were happy with the system and believed that the transition to Mobenzi supported their tasks and functions. They regarded it as a significant change in the way they used to record information in the field. The mHealth application generated electronic forms that made data entry easier, and it was similar to what they used previously with paper-based documentation. However, they also expressed that it would be ideal if the users of any innovation were involved in the selection and implementation of digital innovations.

“*With Mobenzi it was easy because the forms were transferred into the phone software*, *we didn’t have to carry heavy files”* ***P6***

“*The Mobenzi supported our work because the design and content was similar to the forms that we used*, *the electronic templates made data management easier*.*”* ***TL2***

“*When new systems are introduced*, *it is always better to involve users early on*, *or allow us chance to give feedback on the system before it is fully implemented”* ***TL3***

*Training and technical support*. All participants indicated that they received adequate training on the basic functionalities of the mobile system and the operational processes. The majority indicated that the system was not complicated but easy to use. A few found it difficult at first, but they got help from their peers. OTLs agreed and revealed that they were adequately trained on all features after their tablets were loaded with Mobenzi software. They progressively gained confidence as they used the application. OTLs also expressed that CHWs learned faster than they did, and discovered additional features of the application’s functionalities.

“*Our team received training in the application and the technicians were with us to give support*, *as team leaders*, *we provided support to the CHWs*, *we taught them how to operate the system*, *but they learned faster*, *they discovered many things that we did not know*. *Implementing Mobenzi was good for us”* ***TL4***

*“When we started with Mobenzi it was difficult, but I was helped by my colleagues and within a week, I had mastered the gadget” **P10***

*Improving flow of communication*. Participants understood that collaboration between WBOTs was critical, as CHWs were mostly out in the communities by themselves. They believed Mobenzi was implemented to link the CHWs and OTLs effectively. The platform made it easier for them to communicate, support each other, and share the data collected during home visits. They lamented that they could not take images of conditions they encountered and transmit these real-time to their team leaders. The majority of CHWs believed this feature could enhance their effectiveness in managing clients in the communities.

*“During the day we communicated by WhatsApp. You could chat with your colleague and say ‘I have a problem at this house number’. Every ward had group chat for support, we could also chat to team leaders to seek support or help. It would have better if we used cameras to send pictures to the team leaders for additional support” **P5***

#### Theme 2: Using the application

*Effective monitoring and supervision*. OTLs claimed that Mobenzi was beneficial in providing critical support and monitoring the activities of the CHWs. The information the CHWs entered into their phones was relayed in real-time to their tablets. Through the GPS system, OTLs were able to track the households visited, as well as the location and duration of the visit. The system simplified their task and also reduced the travel time for supervision visits. Therefore, monitoring was done on a regular basis. Pre-fieldwork meetings with the CHWs, and in some instances, post-fieldwork meetings for planning, were also facilitated through the system.

“*Mobenzi was brought in to be the eyes and ears for us*. *It was possible to see who was performing*, *and who was malingering and everything*. *When you have allocated your CHWs and you want to check their home visits*, *the GPS would show you that B3 has been to this household and she has done 1*, *2*, *and 3*. *Weekly or monthly*, *you could also monitor their aggregated activities”* ***TL2***

“*Pop-up alerts or messaged enabled us to see the CHW who needed help and sometimes*, *when they were faced with an emergency situation*, *you could immediately call for an ambulance if you could not go”* ***TL1***

*Decision support system*. The participants indicated that Mobenzi supported their activities as stipulated in community health care guidelines. The majority emphasised that it was easier to capture and register households’ information, keep track of the number of registered clients within the households, update households’ information in case of new family members, automatically schedule home visits, and identify vulnerable households for referral to clinics and relevant service providers. The alerts or reminders for missed visits, embedded health education content, as well as the registration of pregnant women, were perceived as the best functionalities of the system. Mobenzi also calculated pregnant women’s expected date of delivery (EDD), offered additional pop-up questions during history taking, and scheduled visits according to the client’s risk profile.

“*First we registered the household using the electronic forms in the phone*. *That helped us to identify who was in need for a referral*, *who had issues with adherence*, *under five and pregnant women*. *The automatic scheduling*, *built-in questions and reminders supported our activities perfectly”* ***P20***

Participants acknowledged that the application assisted them in compiling monthly reports. Their work was easier because the system organised their daily work, automatically summarised all information captured, and generated monthly reports that were available for OTLs in real-time. OTLs entered the community health information collected from CHWs into the DHIS. The system also conveniently had different built-in forms for under-fives, adults, TB adherence and pregnancy. Participants believed privacy was ensured as they had to log in to the system, thus the risk of unauthorised access to clients’ information was minimal.

“*Mobenzi will summarize any information immediately*, *it kept one organized*. *Month-end*, *you got total households visited*, *you just press the button and get various reports*, *for pregnancy*, *post-natal*, *under-fives etc…”* ***P5***

“*We need to compile data and entering them into DHIS*. *Mobenzi was a tracker and a quick reporter”* ***TL1***

Findings revealed that the application was useful because it assisted participants in managing data effectively, with no chance of data entry errors. The application had offline functionality that enabled them to retrieve, search for and enter the required information. It also allowed them to retrieve previous months’ statistics.

“*Mobenzi enabled us to collect complete and accurate community and family health information”* ***P23***

“*When you want stats for ANC*, *under5*, *chronic etc*., *one could retrieve previous work to check and compare statistics”* ***TL2***

*Challenges experienced*. The participants reported several technical and social barriers that possibly hindered the rollout of the system. They were all aware that the phones were programmed and attached to specific people. When they used a refurbished device, it was sometimes difficult for the system to change over to the new CHW in the new ward. In other instances, it failed to report their daily progress and notifications, and declined passwords. The most challenging issue was the system’s occasional failure to report households’ registration. A few participants also reported slow responses in registrations and back-referrals.

“*Mobenzi gave me problems at times*, *because when I was looking for notifications*, *they wouldn’t be there*, *only to appear the following day or after 2 days*. *At times*, *log in was problematic”* ***P1***

The speed of the application tended to be slow, and errors sometimes occurred when they captured information. Some participants reported that it was difficult to retrieve lost data, delete unwanted household information, or add new households and update new information using the application. Sometimes data would randomly disappear from the system, or the system would not allow them to save data without internet connectivity.

“*Another problem was that when a family has relocated and you want to delete them and put on new ones*, *it would not do that*. *When you visit the new household*, *the relocated households’ information would be re- appearing on the phone”* ***P15***

“*At times*, *some households would be deleted from your phone then reappear later”* ***P10***

The size of the screen and keypads created some data entry challenges. Poor signal strength and the weak battery life of their devices were also mentioned. However, the battery lifespan was not the same for every device. One participant also reported the loss of captured data for some months.

“*Another challenge was that the battery was weak”* ***TL3***

“*These batteries were not the same for everybody*. *Some*, *after charging for the whole night would last for the whole day*. *Others did not last*. *They would become flat during the day”* ***P18***

The participants mentioned that most technical problems were addressed by a technician situated in a remote location, who sometimes took a week or even a month to attend to problems. They felt a significant absence of close engagement with Mobenzi’s staff to listen to feedback and suggestions from them, as users of the system. This caused some delays in executing their tasks.

“*I once had to go for the whole month with my phone not working*. *The OTL had to phone Cape Town and wait for a week for the response*. *The problem was*, *we did not have a relevant person within the district to attend to such problems*. *All problems were solved in Cape Town and this took a long time”* ***P23***

#### Theme 3: Reverting to paper-based documentation

*Response to termination of mHealth*. WBOTs explained that the Department of Health discontinued the mHealth application, and they had no prior knowledge of the events or reasons leading to the termination. The return to paper-based recording was demotivating because it meant going back to tedious paper documentation. CHWs explained that the reversion was not easy because it affected their daily performance. They believed they were less likely to succeed because they had multiple roles and numerous forms to complete per household.

“*The withdrawal of Mobenzi was a violation of worker’s rights*. *They snatched off my support system*, *nothing is the same”* ***P11***

“*This is difficult for us*, *everything that we do is affected*.*”* ***P9***

*Impact of paper-based documentation*. Participants found the capturing of data challenging and burdensome. There is prolonged documentation using multiple forms, and it is time-consuming to complete the required paperwork at the end of the month. Data errors are common, especially in light of the number of households allocated to each CHW. The different types of forms required are not always available, and the potential for missing data is high.

“*The papers stay in the bag*, *so they get crumpled and untidy*.*”* ***P5***

“*With paperwork*, *it is difficult to maintain records and to keep them safe at times*, *sometimes we forget the correct documents or they are not available*, *or the patient cannot sign*.*”* ***P18***

OTLs further explained that there is a high risk of capturing inaccurate weekly and monthly summaries into the DHIS.

“*When it comes to reporting at the end of the month*, *we experience data quality problems”* ***TL3***

“*So with paper it’s difficult*. *I spend more than 45 minutes*. *When going to visit households*, *I must carry maternity records*, *individual records*, *tick sheets*, *TB records etc*. *Mobenzi had all the forms built-in”* ***P1***

“*We often hear of documents that got lost*, *get incomplete registration and referrals forms*.*”* ***TL4***

## Discussion

The qualitative, exploratory, descriptive research method was suitable for this study because it provided an in-depth, holistic, contextual, and sensitive understanding of mHealth’s implementation. The method relies heavily on individuals (population) who offer rich accounts of their experiences.

In this case, the qualitative research approach was relevant since the study’s findings reflected WBOTs’ views and experiences of the mHealth initiative in the province, the effectiveness of Mobenzi, its ease of use, and their disappointment in the termination of the programme. Detailed views on support for registrations, the transmission of patient information to OTLs, monitoring and health promotion for individuals and communities were presented.

This mobile application was a pilot that was never scaled, and questions remained about the implementation of digital innovations in resource-constrained contexts. To that end, the exploratory design was found to be beneficial [22] for this study, as it allowed the researchers to develop open-ended questions to which participants freely shared in-depth information. WBOTs provided their insights into mHealth’s implementation in the province, and very little information was available on WBOTs’ experiences prior to this study.

The study’s findings indicate that generally, the WBOTs understood the need for a supportive technological application. They welcomed the transformation in how they managed community-based data and were satisfied with the implementation of the mHealth application (Mobenzi). They found it easy to use and it supported their day-to-day tasks adequately. Participants reported it was a relief from the tedious paper-based data capturing. The digital content design made it simple for them to use because it was modelled on the paper forms they used previously. However, the fact that the Ministry of Health excluded WBOTs in the decision to purchase the technology was not well-received. Participants believed that users should be involved in decisions about the applications they are meant to use.

According to Mwendwa, when staff are introduced to a new system, standard training and documentation should be given to all users to ensure they can operate it effectively [23]. The teams received training and were provided with technical assistance with regard to the basic functionalities and operational processes of the mobile system. Most felt the training was adequate. Those who found it difficult, asked peers to support them and were then able to fully utilise its functionalities. Others were technically savvy and discovered some functionalities on their own; such evidence of the users’ creativity makes a compelling argument for health officials to invest in sustainable digital initiatives for community-based care. Participants lamented the distance between them and the technical teams and the fact that they could not communicate directly with the technicians.

CHWs work in communities away from their team leaders, and the application’s real-time communication and support allowed them to handle their clients effectively. It is evident that the application enabled them to share data easily, seek advice and request emergency support without any delays. These findings are consistent with Chib, Lwin, Ang, Lin and Santoso’s [24] assertions that mobile phones are social enablers as they allow users to build and maintain relationships. Findings showed that communication and workflow were adequately supported, and the built-in chat function enabled good communication with peers and team leaders. The CHWs, as the majority of participants in the teams, expressed that the mobile devices and the application represented a significant opportunity for them to contribute to improving the health outcomes of the communities.

OTLs claimed that the system simplified their role of providing remote supervision to CHWs, and reduced the travel time for supervision visits. The emphasis on a formal evaluation of CHWs suggests that OTLs believed the mobile intervention’s success rest on proper supervision to generate adequate data on the effectiveness of these technologies in community-based settings. They were particularly satisfied with the GPS functionality, which enabled them to track the site and duration of each visit.

Data show that the mobile application met users’ expectations in its ability to collect field-based health data, receive alerts and reminders, facilitate health education sessions to detect and prevent non-communicable diseases, provide ante-natal services, and follow up to ensure enhanced access to health care. The application supported CHWs to register households and clients faster, identified those who needed referrals quickly, the system organised their daily tasks, and automatically summarised all information captured. It also generated weekly and monthly reports that were transmitted real-time to OTLs for entry into the DHIS. This application was designed with custom-made electronic forms for data management [5], and the adoption of the application represented a significant shift in community-based care and community-based health information management. The offline functionality was viewed favourably, especially in rural settings where continuity of power or signal strength were not always guaranteed. This functionality allowed WBOTs to retrieve previous data and make comparisons when they evaluated their performance.

The increase in the number of mobile phone users and mobile applications’ usability is becoming critical in strengthening health care [12]. Mobile health technologies have been found useful in extending care to rural communities. This province embraced innovation early on as it was among the few regions that piloted the mHealth system [7]; the mobile application intended to strengthen the teams and support their tasks [5].

Participants also reported some challenges with the system. Most noted were technical issues such as data that went missing, the device’s poor battery life and size of the screens, notification systems and problems with recycling phones. When CHWs are recruited, they are linked to a specific phone with a particular community profile in the respective wards. A few participants mentioned incorrect households appearing on their “inherited” phones, and it would appear that this was a technical glitch in reprogramming the phone to capture the new user’s community profile. The issue of accessible technical support was also emphasised, as it would eliminate delays in having the system restored. Leon, et al. argue that a locally based technician, with adequate skills and reliable technical support, could assist in updating and maintaining the device each time a need arises [5]. The difficulties participants experienced with downloading speeds could have been caused by low connectivity, as is often the case in rural areas. Despite these challenges, the teams were satisfied with the application.

### Significance of mHealth applications

Neupane, et al. indicated that CHWs provide preventive health services, improve access to basic health care services, collect health-related data, monitor the community’s health, and act as a link between the community and the health system. mHealth applications for WBOTs allow the OTLs to instantly review the information captured by CHWs and enable them to provide timely feedback [25].

Tomlinson, et al’s study found that, in South Africa, mobile phone-based information system platforms offer significant opportunities to improve CHW-delivered interventions [26]. Confirming the findings, Thondoo, et al. [27] determined that the advent of mHealth presents an opportunity to improve support for CHWs. It allows CHWs to send data to a central information system, and data can also be sent from a central location to the periphery. OTLs’ immediate access to CHWs’ data leads to timely identification and control of any disease outbreaks, as well as more efficient long-term surveillance of endemic conditions in health programmes [28]. The most satisfying features of the Mobenzi application were the reminder, automatic scheduling of visits based on clients’ health profiles, and health education functionalities.

The mHealth initiative was suspended after being piloted for almost three years, signalling the return to paper-based recording. It seemed that the Mobenzi programme was withdrawn from the participants’ area of operation without adequate communication from the health authorities. This led to dissatisfaction and unhappiness among CHWs, because it meant a return to substantial paper-based documentation, potentially resulting in reduced quality of health data. Evidence suggests that mobile technology presents opportunities to improve the range and quality of services provided by CHWs [29]. Currently, CHWs appear to spend more time on documentation rather than providing care to communities. Moreover, poor data quality is mainly attributed to document loss, incomplete registration forms, and incomplete referrals. Studies show that combining CHWs’ programme with mHealth is an avenue that could improve the quality of community health data and, subsequently, community-based health services [30, 31]. A mismatch between the number of activities CHWs performed and their documentation might affect the effective implementation of PHC and possibly result in poor health outcomes.

## Limitations

The following limitations were noted in this study. Data were collected immediately after the termination of the mHealth initiative, and there could have been memory lapses and some bias. However, two focus group discussions were conducted to mitigate such risks. The second focus group provided an opportunity to validate the findings of the first group. Due to geographic distance, the researchers did not go back to North West province to discuss the findings with the participants.

## Conclusions

Insights from this study presented a preliminary assessment of the mHealth applications used by WBOTs in one province that took the initiative to pilot the technology. Ultimately, the application was well-received by the community-based teams. They perceived Mobenzi as a functional software application, which allowed them to effectively and efficiently participate in PHC initiatives. The knowledge generated from this study provides an understanding of WBOTs’ experiences regarding the value of mobile technologies, which can inform future mHealth plans in the province and across South Africa. The researchers believe that this understanding can contribute to the broader discourse on the implementation of mHealth programmes in resource-constrained settings. The findings can be of value to the National Department of Health (NDoH) to evaluate whether the designed mHealth programme was effective in integrating the PHC system in terms of coordinating and promoting smart communication. It can also be determined whether it is an effective data collection system, offers decision-making support, and acts as a communication enhancer between CHWs, OTLs and health facility staff.

Findings from this study may provide insights for the NDoH regarding the design and functionalities of mHealth applications they can adapt to optimise their success. The termination of Mobenzi created tensions and anxieties among CHWs, and the flow of communication between the health system/department and teams was not ideal. Such disruptive changes seemed to have been overlooked, yet their impact on the quality of community-based data was evident. mHealth has been proposed as a potential solution to resource constraints, poor access to health care services, data-driven decisions, and similar challenges in developing countries.

It can be concluded that a community-based health care programme that uses WBOTs and mHealth technologies is essential in strengthening community-based health information management in PHC. However, for countries still grappling with decisions on the most appropriate and affordable mobile technologies, there is a need for vigorous evaluation of technology, human and organisational issues prior to implementation. The main features of the technology should be developed with the involvement of users to satisfy their needs. The interventions must be developed with a clear plan regarding expected outcomes, connectivity and technical support. studies need to be conducted to evaluate benefits to PHC. At the time of writing this paper, the province had introduced another type of mHealth technology for the teams; the researchers hope these findings will be useful to authorities to ensure its sustainability.

## Supporting information

S1 Table Interview guide. (DOCX)

S2 Table Checklist. (DOCX)

S1 File Coding tree. (DOCX)

## References

[1] Department of Health. Provincial guidelines for the implementation of the three streams of PHC re-engineering; 2011. Available from: http://policyresearch.limpopo.gov.za/bitstream/handle/123456789/882/Provincial%20Guidelines%20for%20the%20implementation%20of%20the%20three%20Streams%20of%20PHC%20Re-engineering.pdf?sequence=1

[2] K Le Roux, Le Roux I, Mbewu N, Davis E. The role of community health workers in the re-engineering of primary health care in rural Eastern Cape. South African Family Practice. 2015; 57(2): 116–120. Available from: http://www.ncbi.nlm.nih.gov/articles doi: 10.1080/20786190.2014.977063 26279948 PMC4532349

[3] Rikhotso RH. The challenges of Community Development Workers in the implementation of the Community Development Workers’ Programme in Makhado Local Municipality, Limpopo Province. Master of Public Administration. Stellenbosch: University of Stellenbosch; 2013. Available from: https://scholar.sun.ac.za/bitstream/handle/10019.1/85656/rikhotso_challenges_2013.pdf?sequence=1

[4] Maes K, Kalafonos I. Becoming and remaining community health workers: Perspectives from Ethiopia and Mozambique. Social Science & Medicine. 2013; 87(1): 52–59. Available from: https://www.ncbi.nlm.nih.gov/pubmed/23631778] doi: 10.1016/j.socscimed.2013.03.026 23631778 PMC3732583

[5] Leon N, Schneider H, Daviaud E. Applying a framework for assessing the health system challenges to scaling up mHealth in South Africa. Medical Informatics and Decision Making. 2012; 12(1): 1–12. Available from: http://www.biomedcentral.com/1472-6947/12/123 doi: 10.1186/1472-6947-12-123 23126370 PMC3534437

[6] Mobenzi. Mobile Technology to Empower Community Healthcare Workers (CHWs) and Inform Decision-makers; 2017. Available from: http://www.mobenzi.com/docs/CHW%20Info%20Pack%20-%20May%202017.pdf

[7] Assegaai T, Reagon G, Schneider R. Evaluating the effect of ward-based outreach teams on primary healthcare performance in North West Province, South Africa: A plausibility design using routine data. South African Medical Journal. 2018; 108(4): 329–335. Available from: https://www.ncbi.nlm.nih.gov/pubmed/29629685 doi: 10.7196/SAMJ.2017.v108i4.12755 29629685

[8] Odendaal WA, Lewin S. The provision of TB and HIV/AIDS treatment support by lay health workers in South Africa: a time-and-motion study. Human Resources for Health. 2014; 12(18): 1–7. Available from: https://human-resources-health.biomedcentral.com/articles/10.1186/1478-4491-12-18 doi: 10.1186/1478-4491-12-18 24708871 PMC3978134

[9] Botha A, Booi V. mHealth Implementation in South Africa. International Information Management Corporation. 2016; (1): 1–13. Available from: https://www.researchgate.net/publication/303563981_mHealth_Implementation_in_South_Africa

[10] Ojo AI. mHealth interventions in South Africa: A review. Durban: SAGE Publishers; 2018. Available from: https://journals.sagepub.com/doi/pdf/10.1177/2158244018767223

[11] National Department of Health. (NDoH). MHealth Strategy South Africa 2015–2019. Pretoria: National Department of Health; 2015. Available from: http://www.hst.org.za/publications/NonHST%20Publications/mHealth%20Strategy%202015.pdf

[12] Wesson JL, Singh A, van Tonder B. Can adaptive interfaces improve the usability of mobile applications?. Department of computing sciences: Nelson Mandela Metropolitan University; 2010. Available from: https://www.researchgate.net/publication/225261962_Can_Adaptive_Interfaces_Improve_the_Usability_of_Mobile_Applications

[13] Modi K, Mohanty RB. Mhealth: Challenges, benefits and Keys to successful implementation. Bangalore India: Infosys limited; 2015.

[14] Leon N, Schneider H. MHealth4CBS IN South Africa: A review of the role of mobile phone technology for monitoring and evaluation of community-based health services. Cape Town: University of Western Cape; 2012. Available from: http://www.hst.org.za/publications/NonHST%20Publications/MHealth4CBS-A%20Review.pdf

[15] Local Government. Dr Kenneth Kaunda District Municipality; 2017. Available from: https://municipalities.co.za/overview/140/dr-kenneth-kaunda-district-municipality

[16] Streubert H, Speziale HJ, Carpenter DR. Qualitative research in nursing: advancing the humanistic imperative. 5^th^ edition. Philadelphia: Lippincott Williams & Wilkins; 2011. ISBN: 0781796008.

[17] Babbie ER. 2016. The practice of social research. 14^th^ edition. Chapman University: Cengage Learning; 2016. ISBN: 1305445562.

[18] Qalinge L. Community home-based care programme: a marginalised key community resource. Social Work. 2011; 47(1): 51–58. Available from: http://socialwork.journals.ac.za/pub/article/view/142

[19] Tappen RM. Advanced Nursing Research: From theory to practice. 2^nd^ edition. USA: Jones & Bartlett Learning; 2016. ISBN 9781284048308.

[20] Silverman RM, Patterson KL. Qualitative Research Methods for community development. New York: Taylor & Francis; 2015. ISBN: 0415740363.

[21] Liamputtong P. Qualitative research methods. 4^th^ edition. Victoria: Oxford University Press; 2013. ISBN: 0195518551.

[22] Royce D. Research methods in Social Work. 5^th^ edition. USA: Thompsons Learning Inc; 2008. ISBN: 1516507185.

[23] Mwendwa P. What encourages community health workers to use mobile technologies for health interventions? Emerging lessons from rural Rwanda. Development Policy Review. 2016; 36(1): 111–129. Available from: https://ideas.repec.org/a/bla/devpol/v36y2018i1p111-129.html

[24] Chib A, Lwin MO, Ang J, Lin H, Santoso F. Midwives and mobiles: using ICTs to improve healthcare in Aceh Besar. Indonesia, Asian Journal of Communication. 2008; 18(4): 348–364. Available from: 10.1080/01292980802344182

[25] Neupane S, Odendaal W, Friedman I, Jassat W, Schneider H, Doherty T. Comparing a paper-based monitoring and evaluation system to a mHealth system to support the national community health worker programme, South Africa: An evaluation. BMC Medical Informatics and Decision Making. 2014; 14(69): 1–9. Available from: http://www.biomedcentral.com/1472-6947/14/69 doi: 10.1186/1472-6947-14-69 25106499 PMC4150556

[26] Tomlinson M, Rotheram-Borus MJ, Doherty T, Swendeman D, Tsai AC, Ijumba P, et al. Value of a mobile information system to improve quality of care by community health workers. SA Journal of Information Management. 2013; 15(1): 1–9. Available from: https://www.ncbi.nlm.nih.gov/pubmed/25147730 doi: 10.4102/sajim.v15i1.528 25147730 PMC4137890

[27] Thondoo M, Strachan DL, Nakirunda M, Ndima S, Muiambo A, Källander K, et al. Potential Roles of mHealth for Community Health Workers: Formative Research with End Users in Uganda and Mozambique. JMIR mHealth and uHealth. 2015; 3(3): 1–8. Available from: https://www.ncbi.nlm.nih.gov/pubmed/26206419 doi: 10.2196/mhealth.4208 26206419 PMC4528084

[28] Kahn JG, Yang JS, Kahn JS. Mobile Health needs and opportunities in developing countries. Cell phones and mHealth. 2010; 29(2): 254–261. Available from: https://www.ncbi.nlm.nih.gov/pubmed doi: 10.1377/hlthaff.2009.0965 20348069

[29] Braun R, Catalani C, Wimbush J, Israelski D. Community Health Workers and Mobile Technology: A Systematic Review of the Literature. Community Health Workers and Mobile Technology. 2013; 8(6): 1–9. Available from: https://journals.plos.org/plosone/article doi: 10.1371/journal.pone.0065772 23776544 PMC3680423

[30] Schuttner L, Sindano N, Theis M, Zue C, Joseph J, Chilengi R, et al. A Mobile Phone-Based, Community Health Worker Program for Referral, Follow-Up, and Service Outreach in Rural Zambia: Outcomes and Overview. Telemedicine and eHealth. 2014; 20(8): 721–728. Available from: https://www.ncbi.nlm.nih.gov/pubmed/24926815 10.1089/tmj.2013.0240 PMC4106387 24926815

[31] Vision World. Voices from the Field: Community Health Workers’ experience with mHealth; 2018. Available from: https://www.wvi.org/sites/default/files/CHW%20mHealth%20story_FINAL_pdf_2.pdf

