# Supplementary material for: Implementation of mHealth applications in community-based health care: Insights from Ward-Based Outreach Teams in South Africa
Source: PLoS One. 2022 Jan 25;17(1):e0262842. doi: 10.1371/journal.pone.0262842 (PMC8789095; doi:10.1371/journal.pone.0262842)
Supplement: S1 Table — (DOCX) [file pone.0262842.s001.docx]

# **S1: Table**

# **Interview Guide**

**SECTION A: BIOGRAPHICAL DATA**

1. Gender ______________________

2. Age____________________

3. What is the type of position you hold in WBOTs? ___________

4. How long did you use Mobenzi (in months)? ________________

**SECTION B: QUESTIONS**

| **Focus area of questioning** | **Rationale** |
| --- | --- |
| **WBOT Program**  Tell us about your work as Outreach teams in this district *(Opening question).*   - How are you linked to the local health facilities? - What is your main role? Community health workers/Team leaders? - How does the department of health support your work? | To gain deeper understanding of the:   - context, structure and functioning of WBOTs within PHC. - implementation of CHWs policy in the district - support by the District Management Team mandated with establishment of CHWs program |
| **mHealth used in the province**   - What are your views regarding the mHealth implementation in North West Province? - Can you describe the earlier systems that you used? - How was Mobenzi introduced? - What type of training did you receive? Who trained you? Content? - What do you think of the technical support you received? | - Exploring the evolution of mHealth in the province, district and sub-district - Gaining an understanding of the processes the district implemented in the introduction of the mobile technology - Preparations for the implementation, including capacity building initiatives - Involvement of users in the selection of the technology |
| **mHealth and WBOT roles**   - How did Mobenzi support your tasks in the community? Supporting role (Team leaders)? - Please share your experiences with registration of households using Mobenzi - What did you like best about the application? - What did you like least? | - Exploring views regarding usability of the application - Specific tasks and support provided by the application - Challenges experienced - Solutions they created - Support from the district - Recommendations for future applications |
| **Termination of the application**   - How was the application terminated? - How was the decision communicated to you? - What was your reaction? - How do you manage community health data currently? - Challenges in maintaining accurate data? Timeous reporting? Completeness of data? - What do you think should be done? | - Decision making systems/involvement of users - Plans put in place for termination - Impact of paper-based reporting on data quality - Recommendations to improve data quality |
| - Is there anything else you would like to share? (*Final question*) | |
| NB: Probing will be based on responses. | |
