# Supplementary material for: Implementation of mHealth applications in community-based health care: Insights from Ward-Based Outreach Teams in South Africa
Source: PLoS One. 2022 Jan 25;17(1):e0262842. doi: 10.1371/journal.pone.0262842 (PMC8789095; doi:10.1371/journal.pone.0262842)
Supplement: S2 Table — (DOCX) [file pone.0262842.s002.docx]

**S2 Checklist**

**Consolidated criteria for reporting qualitative studies (COREQ): mHealth manuscript.**

**32-item checklist**

Table 2: Supplementary file

| No. item | Guide question/description | Location in manuscript# |
| --- | --- | --- |
| **Domain 1: Research team reflexivity** |  |  |
| *Personal characteristics* |  |  |
| 1. Interviewer facilitator. | Which authors conducted the focus groups? | Methods *(p6,7)* |
| 1. Credentials. | What were the researchers credentials? | RST graduate student.  MMR joint project lead investigator (PhD) *(p1)* |
| 1. Occupation. | What was their occupation at the time of the study? | Methods *(p6)* |
| 1. Gender. | Was the research male or female? | Females |
| 1. Experience and training. | What experience or training did the researchers have? | RST graduate student.  MMR joint project lead investigator (PhD) *(p6)* |
| *Relationship with participants* |  |  |
| 1. Relationship established | Was a relationship established prior to study commencement? | N/A |
| 1. Participant knowledge of the interviewer | What did the participants know about the researcher? e.g. personal goals, reasons for doing the research | RST disclosed her student status *(p6)* |
| 1. Interviewer   characteristics | What characteristics were reported about the inter viewer/facilitator? e.g. Bias,  assumptions, reasons and interests in the research topic | No biases were identified. The paper is part of the bigger mHealth study implemented in 4 provinces in South Africa *(p4,7)* |
| **Domain 2: study design** |  |  |
| *Theoretical framework* |  |  |
| 1. Methodological   orientation and  Theory | What methodological orientation was stated to underpin the study? e.g. grounded theory, discourse analysis, ethnography, phenomenology, content analysis | Discussion. Used as justification*. (p16*) |
| *Participants selection* |  |  |
| 1. Sampling | How were participants selected? e.g. purposive,  convenience, consecutive,  snowball | Methods *(p5,6)* |
| 1. Method of approach | How were participants approached? e.g.  face-to-face, telephone, mail, email | Methods. WBOTs Manager assisted with recruitment. *(p5,6)* |
| 1. Sample size | How many participants were in the study? | Results *(p8)* |
| 1. Non-participation | How many  people refused to participate or  dropped out? Reasons? | Methods *(p8)* |
| *Setting* |  |  |
| 1. Setting of data collection | Where was the data collected? e.g. home, clinic, workplace | Setting *(p5)* |
| 15. Presence of non  -participants | Was anyone else present besides the participants and researchers? | Results *(p8)* |
| 1. Description of sample | what are the important characteristics of the sample?  e.g. demographic data, date | Results *(p8,9)* |
| *Data collection* |  |  |
| 1. Interview guide | Were questions, prompts, guides provided by the authors? Was it pilot tested? | Methods *(6,7)* |
| 1. Repeat interviews | Were repeat inter views carried out? If yes, how many? | N/A |
| 1. Audio/visual | Did the research use audio or visual recording to collect the data? | Methods *(p7)* |
| 1. Field notes | Were field notes made  during and/or after  the interview or focus group? | Methods *(p7)* |
| 1. Duration | What was the duration of the inter views or focus group? | Methods *(p7)* |
| 1. Data saturation | Was data saturation discussed? | Methods |
| 1. Transcripts returned | Were transcripts returned to  participants for comment and/or correction? | N/A |
| **Data analysis and findings** |  |  |
| *Data analysis* |  |  |
| 1. Number of coders | How many data coders coded the data? | Methods *(p8)* |
| 1. Description of the coding tree | Did authors provide a description of the coding tree? | Coding tree annexure attached. *(p8)*  Provided a table with themes, sub-themes and categories  Themes derived from data. *(p9)* |
| 1. Derivation of themes | Were themes identified in advance or  derived from the data? | Methods *(p8,9)* |
| 1. Software | What software, if applicable, was used to manage the data? | Manual *(p8)* |
| 1. Participant checking | Did participants provide feedback on the findings | Limitations *(19)* |
| *Reporting* |  |  |
| 1. Quotations presented | Were participant quotations presented to illustrate the themes/findings? Was each  quotation identified? e.g. participant number | Results. Used participant numbers. *(p9 to 16)* |
| 1. Data and findings consistent | Was there consistency between the data presented and the findings? | There is consistency between data and findings *(p8)* |
| 1. Clarity of major findings | Were major themes clearly presented in the findings? | Results. Table 1. *(p9)* |
| 1. Clarity of minor findings | Is there a description of diverse cases or discussion of minor themes? | Results. Table 1: Theme 3. |
