# Supplementary material for: Implementation of mHealth applications in community-based health care: Insights from Ward-Based Outreach Teams in South Africa
Source: PLoS One. 2022 Jan 25;17(1):e0262842. doi: 10.1371/journal.pone.0262842 (PMC8789095; doi:10.1371/journal.pone.0262842)
Supplement: S1 File — (DOCX) [file pone.0262842.s003.docx]

mHealth implementation in North West Province

Transition from paper to e-system

Using Mobenzi

Reverting to paper-based documentation

Evolution of Mobenzi

Capacity building

Experiences of Mobenzi

Initial reaction

Data management diffculties

Improve data capture

Welcomed change

Easy recording

Familiarity with reporting forms

Enhance participation

Support mechanisms

- Happy with training
- Real time

Communication

Peer support

High confidence

Household registration

Automatic Scheduling

Risk assessment

Referrals

Monitoring - Monthly reports

Tracking – use of DHIS

Alert system

Security of data

Real time interventions by OTLs

Successes of mHealth

User-phone registration

Passwords

Missing data

Poor signal

Deleting obsolete data

Interface issues

Battery life

Availability of technicians

Demotivation

Lack of information

Anxiety

Rights violated

No confidence in data quality

Poor tracing

Prolonged documentation

Poor data quality

Inadequate support

No self monitoring

Data loss

Challenges
